# Supplementary material for: Unconsciousness and amnesia after cross-body electric shocks not involving the head–A prospective cohort study
Source: PLoS One. 2023 Apr 4;18(4):e0283957. doi: 10.1371/journal.pone.0283957 (PMC10072343; doi:10.1371/journal.pone.0283957)
Supplement: Attachment — Submitted filename: Response to reviewers.docx [file pone.0283957.s001.docx]

*Dear Editor and reviewers,*

*Thank you very much for valuable corrections, comments and suggestions.*

*We have now revised the manuscript according to the reviews and specific answers to each point are presented below. We hope you find the provided changes and answers satisfactory.*

*On behalf of the authors*

*Karin Biering*

Review Comments to the Author

**Reviewer #1:** The authors address in a satisfactory way the gap of the examined issue at the introduction section but they should clearly refer the aim of their study at the last paragraph as well as the hypothesis in different sentence.

*Authors reply:* *This is now rewritten to highlight the aim and hypothesis clearly*

The methodology design (experimental tests, measurements etc) needs more details. The methodology has important details missing. Subsection for materials and methodology could help the flow.

*Authors reply: We have added these subheadings and provided more detail regarding the study, that we originally left out to avoid self-plagiarism*

The results of the study are presented in a satisfactory way
The authors should present the main findings of their study at the first paragraph of the discussion section and also compare their findings to similar studies of the literature. Discussion section is to poor. The authors discuss their findings with no other studies.

*Authors reply: We have added the main findings to the beginning of the discussion, and have tried to elaborate the discussion. However, comparing our findings to previous studies is difficult as mentioned by reviewer #2, this is the first study on this topic, so direct comparison is not possible..*

**Reviewer #2**: The manuscript addresses the topic whether immediate CNS symptoms (i.e. unconsciousness, amnesia) might occur in electrical shocks without direct entry or exit point on the head. I appreciate the hypothesis of the study that electric currents flowing through the body and crossing the spinal cord may induce CNS symptoms, even if the head (brain) is not directly affected. This is the first study on this topic. The main strength of the study is the study population because it is based on a prospective cohort of electricians with weekly follow-up.
The data collection and a general description of the shocks are presented in a previous study of the authors including details on the type of electrical shock in terms of current types (AC versus DC) and voltages (low versus high voltage). I recommend to present additionally the numbers for the current types and voltages and to discuss the proportions (e.g., in Table 1) in the present manuscript. Thus, it may be possible to associate the circumstances of the electrical shocks and the outcome further.

*Authors reply: Thank you for these kind words. We have elaborated on the methods section as also reviewer#1 suggested to provide more information regarding the study. We would also have provided more details regarding the circumstances of the shock. However, in when using data from Statistics Denmark it is custom not to report subgroups containing less than 5 persons, so not all details could be reported in the group with affected CNS in table 1. Instead, we have now added a sentence including these characteristics without presenting the exact numbers in the results section*

Additionally, there are some minor points:
Title: ‘a prospective cohort study’ should be added as an important feature of the study.

*Authors reply: This is added to the Title*

The abstract should be structured with the headers ’Introduction’, ‘Material and Methods’, ‘Results’ and ‘Conclusion’.

*Authors reply: The abstract has been provided with the suggested headers*

Line 40: Please provide the risk estimate (i.e. odds ratio (OR).

Line 56: A dot is missing at the end of the sentence.

Line 63: Please add the year in which the study was conducted.
Line 70: Please move the comma to ‘same side’ (non-exposed), ‘opposite side’.
Lines 71-74: These two sentences should be moved to the result section.
Line 83: The risk estimate and confidence interval (95%?) should be provided.
Line 93 and Line 95: Please add the numbers to facilitate reading.
Line 94: A dot is missing at the end of the sentence.
Lines 99: Please delete the dot before the parenthesis.
Lines 101-102 Header of the Table 1: ‘Danish electricians (n=1235)’ is a bit misleading, because 1,235 is the number of events, not of the electricians.

*Authors reply: All of these suggestions has been added/changed*

Table1: There is probably a mistake with the numbers in the table because the four columns of gender, age, severity and exposure do not sum up to 1,235, but to 1,264. Please check it. - In column ‘unconsciousness’ and row ‘somewhat severe or worse’ and ‘Not severe or a little severe’ is an error because the sum is not 100%.

*Authors reply: The numbers are actually correct, but we realize that the presentation of them were not elegant. In the original Table 1, we present three columns of outcome (unconsciousness(n=8), amnesia(n=21) and a combination of unconsciousness and/or amnesia (n=27, two persons reported both outcomes). This means that the last column include all with either unconsciousness or amnesia and those with both. We have made a new layout of the table and added a longer footnote, to make this clear.*

Lines 103 and 112: Is the explanation of ‘Combined’ beneath the table correct or should the logical operator only be ‘and’ instead of ‘and/or’?

*Authors reply: Please refer to our reply regarding table 1 above*

Line 104-107: Please provide the risk estimates and note the non-significant confidence intervals.
Line 105: Please delete the dot before the parenthesis.
Line 109 Table 2: Please add [CI95%] after OR in the second row and add the abbreviation below the table.
Line 114: Please start the discussion with a sentence on the key results (i.e., number of persons and events).
Line 155: Please use ‘Ann N Y Acad Sci.’ to be consistent.

*Authors reply: All of these suggestions has been added/changed*

The manuscript lacks from several punctuation and may benefit from some closer proofreading by a native speaker.

*Authors reply: We have now had professional British proof-reading, thus several minor changes in wording has been changed on top of the suggestions provided by the reviewers.*
